# A Recognition Tag of Human Origin for Bioorthogonal Generation of Antibody‐Drug Conjugates using Microbial Biotin Ligase

**DOI:** 10.1002/cbic.202500261

**Published:** 2025-05-06

**Authors:** Peter Bitsch, Sebastian Bitsch, Noah Murmann, Ingo Bork, Janine Becker, Harald Kolmar

**Affiliations:** ^1^ Clemens‐Schöpf Institute for Organic Chemistry and Biochemistry Technical University of Darmstadt Peter‐Grünberg‐Str. 4 64287 Darmstadt Germany; ^2^ Centre of Synthetic Biology Technical University of Darmstadt Peter‐Grünberg‐Str. 4 64287 Darmstadt Germany

**Keywords:** antibody‐drug conjugates, bioconjugation, biotin ligase, MMAE, p67

## Abstract

The use of enzymes, such as microbial transglutaminase, lipoate protein ligase A, or sortase A, for the generation of antibody‐drug conjugates has proven to be a powerful tool for the site‐specific payload conjugation to tumor‐specific antibodies. Herein, the extension of this enzymatic toolbox by *Pyrococcus horikoshii* biotin ligase is reported. To this end, the therapeutic antibody trastuzumab is equipped with p67, the 67 amino acid carboxyl‐terminal domain of human propionyl‐CoA carboxylase *α* subunit, at the C‐terminus of either the light or heavy chain (Trz‐LC:p67 and Trz‐HC:p67). Upon incubation with PhBL, the azide‐bearing linker desthiobiotin azide is site‐specifically coupled to the p67 domains at the antibody. Subsequent strain‐promoted azide‐alkyne cycloaddition with DBCO‐AF488 and DBCO‐Val‐Cit‐PAB‐MMAE yielded conjugates near to full conversion. In cellular assays, these constructs exhibit single‐digit nanomolar EC50 values in cellular proliferation assays on SK‐BR‐3 and A431 cells, where no significant difference in the performance between the two variants Trz‐LC:p67‐MMAE and Trz‐HC:p67‐MMAE is observed. On high Fc‐γIIIa receptor expressing Jurkat cells, Trz‐HC:p67‐MMAE exhibits higher potency than Trz‐LC:p67‐MMAE, indicating an Fc‐blocking effect of p67 when fused to the light chain.

## Graphical abstract

A human‐derived recognition tag of *Pyrococcus horikoshii* biotin ligase is fused to the monoclonal antibody trastuzumab at the C‐termini of either light or heavy chain. These fusion antibodies allow for the PhBL‐mediated introduction of desthiobiotin azide and the subsequent attachment of DBCO‐AF488 and DBCO‐Val‐Cit‐PAB‐MMAE via strain‐promoted azide‐alkyne cycloaddition. The generated antibody‐drug conjugates exhibit single‐digit nanomolar potency on Her2‐expressing cell lines.

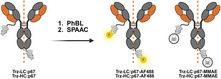

## 1 Introduction

Antibody‐drug conjugates (ADCs) have proven their great potential in targeted treatment of malignancies. Thus, already 13 ADCs achieved clinical approval^[^ ^1^ ^]^ and over 100 are investigated in clinical stages.^[^ ^2^ ^]^ These therapeutics consist of a target‐specific monoclonal antibody (mAb) and a cytotoxic cargo which is attached to the mAb by a molecular linker. Introduction of linker and payload can be conducted in a variety of methods.^[^ ^2^ ^]^ First clinically applied ADCs rely on rather unspecific conjugation of *N*‐hydroxysuccinimidyl‐activated esters to surface‐exposed lysines,^[^ ^3^, ^4^ ^]^ while nowadays toxin conjugation relies on more specific strategies such as introduction of maleimide‐functionalized linker‐toxins to interchain cysteines.^[^ ^1^, ^5^ ^]^ In addition, many efforts have been made to develop chemoenzymatic methods for site‐specific attachment of the cargo to the mAb.^[^ ^5^, ^6^, ^7^ ^]^ These aim to yield more homogenous products that also feature more desirable pharmacokinetic properties, like improved serum stability, while maintaining target selectivity.^[^ ^7^ ^]^

Site‐specific conjugation methods have consequently been linked to the use of enzymes with high substrate specificity. For ADC generation, enzymes, like microbial transglutaminase,^[^ ^8^ ^]^ sortase A,^[^ ^9^ ^]^ formylglycine‐generating enzyme,^[^ ^10^ ^]^ glycosyltransferases,^[^ ^11^ ^]^ prenyltransferases,^[^ ^12^ ^]^ tubulin tyrosine ligase,^[^ ^13^ ^]^ and lipoate protein ligase A,^[^ ^14^ ^]^ have already successfully been employed for the generation of homogeneous ADCs with defined drug‐to‐antibody ratios (DARs).^[^ ^5^, ^15^ ^]^ While most of these ADCs are limited to DARs of 2, since each of the two arms of the symmetric antibody molecules contains a single conjugation site either inside constant antibody domains or at the termini, the implementation of a branched hydrophilic linker system has been shown to allow the chemoenzymatic generation of high‐DAR ADCs with maintained hydrophilic behavior.^[^ ^16^ ^]^

**Figure 1:**
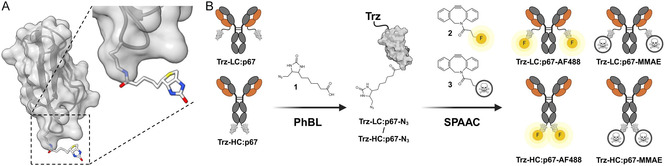
A) p67 biotin acceptor domain assembled from human propionyl‐CoA carboxylase α subunit. Electron microscopic structure (PDB: 8XL5, UCSF ChimeraX).^[^ ^28^ ^]^ B) Design of trastuzumab‐p67 fusion antibodies **Trz‐LC:p67** and **Trz‐HC:p67** and generation of conjugates utilizing PhBL for introduction of desthiobiotin azide **1** and subsequent SPAAC for labeling with DBCO‐AF488 **2** or DBCO‐Val‐Cit‐PAB‐MMAE **3**. Created with Biorender.

Many enzymatic approaches rely on the introduction of recognition sequences to the mAb to enable enzymatic conjugation.^[^ ^5^, ^15^ ^]^ The mostly artificial nature of these tags of nonhuman origin always bears the risk of immunogenic activity when the construct is administered to the patient.^[^ ^17^ ^]^ Thus, enzymes recognizing substrates of human origin would be favorable for the development of novel chemoenzymatic conjugation strategies. Biotin ligases are a class of enzymes, which to the best of our knowledge haven't been investigated for the generation of ADCs in the past. They catalyze the transfer of biotin to a specific lysine within a biotin acceptor domain in form of a two‐step reaction in presence of ATP and along the formation of biotinyl‐adenosine‐monophosphate (biotinyl‐AMP).^[^ ^18^ ^]^ This biotinylation is a rare but specific and essential modification, conducted on only few proteins inside living cells.^[^ ^19^ ^]^ However, due to its chemical properties, biotin cannot directly be used as a linker for covalent attachment of a payload. To circumvent this issue, Slavoff et al. investigated biotin derivatives equipped with functional moieties, such as alkyne and azide groups, and probed their recognition by various biotin ligases to be fused to the 67 amino acid carboxyl‐terminal domain of human propionyl‐CoA carboxylase *α* subunit (p67, **Figure** **1**A).^[^ ^20^ ^]^ Among others, *Pyrococcus horikoshii* biotin ligase (PhBL) favorably accepted desthiobiotin azide as surrogate substrate, thus, enabling subsequent modification of the so functionalized acceptor domain in a second step, for example, with a fluorophore utilizing click chemistry.^[^ ^20^ ^]^

This in mind, the objective of this work was to establish PhBL as an enzyme for the generation of ADCs with a distinct number of payloads using human p67 as a recognition tag. To this end, the anti‐Her2 mAb trastuzumab was selected for modification with the p67 domain at the C‐termini of either the light or heavy chain (Figure 1B), thereby, enabling PhBL recognition and a maximum degree of conjugation of 2. The human origin of p67 could also be beneficial in terms of possible immunogenicity, which can be caused by the introduction of nonhuman tags to antibodies.^[^ ^17^ ^]^ The p67 domain should then allow the biorthogonal introduction of desthiobiotin azide **1** to the mAb. Subsequently, the click‐functionalized cargos DBCO‐AF488 **2** and DBCO‐Val‐Cit‐PAB‐MMAE **3**, a tubulin inhibitor commonly used in frame of ADC generation, could be introduced upon strain‐promoted azide‐alkyne cycloaddition (SPAAC). By labeling of the mAb with AF488, labeling potential of the format could be assessed easily be UV/Vis–spectroscopy, and the generation of an MMAE‐loaded ADC should allow for the evaluation of the format in cellular proliferation assays with classical lysosomal cleavage of the Val‐Cit‐PAB linker for toxin release within the target cell.^[^ ^21^ ^]^

## 2 Results and Discussion

### 2.1 Generation of ADCs

To allow the biotin ligase mediated introduction of **1**, synthesized according to literature,^[^ ^20^ ^]^ two variants of trastuzumab were designed and produced, in which p67 was fused to either the trastuzumab C‐terminus of the light chain (**Trz‐LC:p67**) or the heavy chain (**Trz‐HC:p67**). Trastuzumab is an approved antibody targeting Her2 protein which is overexpressed on the surface of cancer cells and a trastuzumab ADC is approved since 2013 for treatment of breast cancer.^[^ ^1^, ^4^ ^]^ The trastuzumab variant proteins were produced by transient transfection of the respective fusion genes into production cell lines using standard procedures and were >95% pure after protein A purification from the cell culture supernatant. As revealed by size exclusion chromatography (SEC, Figure S1A,B, Supporting Information) the constructs remained monomeric and showed no enhanced aggregation tendency upon amendment of the p67 sequence.

As biotin ligase is expressed in HEK293 cells,^[^ ^22^ ^]^ we investigated whether the produced antibodies were biotinylated to some extent. Indeed, the highly sensitive method of western blot revealed biotinylation of the target proteins to some extent (Figure S2, Supporting Information), but analysis of the antibodies prior to and after additional purification with Strep‐TactinXT 4Flow resin that captures biotinylated proteins, revealed no differences that would suggest significant amounts of biotinylated antibodies (Figure S3A,B, Supporting Information). Thus, for further experiments antibodies were used directly after purification with protein A.

**Figure 2:**
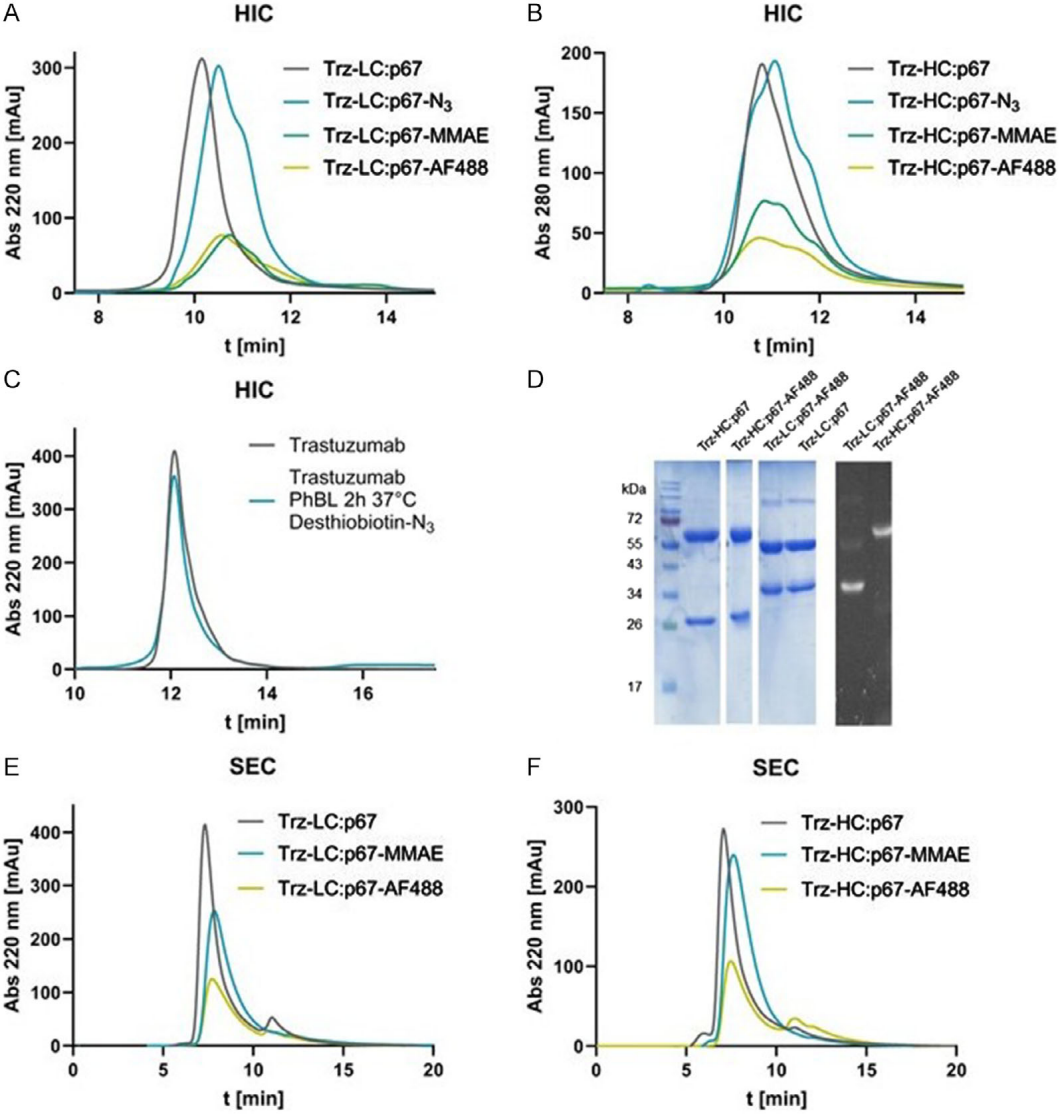
Chromatographic analysis of generated constructs. A) HIC chromatogram of **Trz‐LC:p67** constructs. Note: **Trz‐p67‐N**_**3**_ stands for p67 modified with desthiobiotin azide. B) HIC chromatogram of **Trz‐HC:p67** constructs. C) HIC chromatogram of negative control, Trastuzumab incubated with PhBL, and desthiobiotin azide. D) SDS‐Gel of AF488 conjugates, Coomassie stain, and UV picture. E) SEC chromatogram of **Trz‐LC:p67**‐conjugates. F) SEC chromatogram of **Trz‐HC:p67**‐conjugates.

The introduction of **1** to the antibody variants was conducted upon incubation of mAbs with **1** and PhBL at 37 °C for 3 h. Monitoring of reaction progress by hydrophobic interaction chromatography (HIC) revealed successful conversion of mAbs to **Trz‐LC:p67‐N**_**3**_ and **Trz‐HC:p67‐N**_**3**_ (**Figure** **2**A,B). Subsequently, the desired payload was introduced through SPAAC by incubating **Trz‐LC:p67‐N**_**3**_ and **Trz‐HC:p67‐N**_**3**_ with **2** and **3**, respectively, at 30 °C for a duration of 18 h, yielding **Trz‐LC:p67‐AF488**, **Trz‐HC:p67‐AF488**, **Trz‐LC:p67‐MMAE,** and **Trz‐HC:p67‐MMAE**. HIC monitoring revealed changes in the shape and intensity of the peaks representing the generated constructs, which was consistent across multiple experiments. The introduction of a hydrophobic payload, especially one such as MMAE, was expected to lead to higher retention of the products in HIC. However, the p67 domain seems to hinder hydrophobic interactions between the attached cargo and the column stationary phase. Thus, P67 might also compensate for the hydrophobic impact of the introduced cargos, a feature that would be beneficial for potential applications since increased hydrophobicity of ADCs often correlates with undesired pharmacokinetic properties.^[^ ^23^ ^]^ As a negative control for unspecific enzymatic modification, conventional trastuzumab was treated with the same protocol, revealing no changes in retention time in HIC chromatograms (Figure 2C), therefore, proving specificity of this conjugation method.

The determination of AF488 labeling by UV/Vis spectroscopy revealed labeling ratios of ≈2 for **Trz‐LC:p67‐AF488** and **Trz‐HC:p67‐AF488**. Furthermore, sodium dodecyl sulfate–polyacrylamid gel electrophoresis (SDS‐PAGE) analysis demonstrated the presence of fluorescent bands at the expected molecular weight (Figure 2D). However, a low amount of unspecific labeling was observed, indicated by a low intensity fluorescent band detected for the heavy chain of **Trz‐LC:p67** that contains p67 fused to the light chain. Additional optimization of reaction conditions could lead to further reduction of unspecific modifications of the antibody. The absence of aggregates in postpurification SEC further confirmed the stability of the conjugates (Figure 2E,F).

### 2.2 Cellular Binding Assays

**Figure 3:**
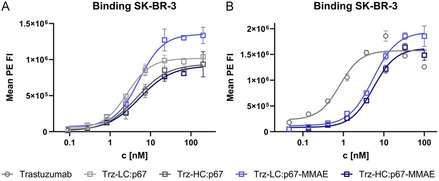
Cellular binding assays on SK‐BR‐3 cells. A) Binding assay of **Trz‐LC:p67**, **Trz‐HC:p67**, **Trz‐LC:p67‐MMAE,** and **Trz‐HC:p67‐MMAE**. B) Binding assay of trastuzumab, **Trz‐LC:p67‐MMAE,** and **Trz‐HC:p67‐MMAE**. Technical triplicates are shown.

The binding properties of trastuzumab, parental trastuzumab‐p67 fusion proteins, and generated conjugates were assessed by on‐cell binding utilizing SK‐BR‐3 cells, which have been shown to exhibit high Her2 expression levels.^[^ ^24^ ^]^ Thus, the binding behavior of unmodified trastuzumab, fusion antibodies **Trz‐LC:p67** and **Trz‐HC:p67**, as well as **Trz‐LC:p67‐MMAE** and **Trz‐HC:p67‐MMAE** was examined upon incubation of cells with the constructs and subsequent staining with an antihuman Fc goat antibody labeled with phycoerythrin. Flow cytometric analysis revealed single‐digit nanomolar EC50 values for the parental antibodies **Trz‐LC:p67** and **Trz‐HC:p67** and the conjugates **Trz‐LC:p67‐MMAE** and **Trz‐HC:p67‐MMAE** (**Figure** **3**A, **Table** **1**). However, as the EC50 value obtained for unmodified trastuzumab (Figure 3B, Table 1) appeared to be significantly lower than the ones obtained for the fusion proteins, the modification of the mAb with p67 was considered to be responsible for slightly impaired cellular binding behavior.

**Table 1:** EC50 values derived from cellular binding assays on SK‐BR‐3 cells. EC50 values of **Trz‐LC:p67‐MMAE** and **Trz‐HC:p67‐MMAE** depicted in the table were derived from Figure 2a.

Construct	EC50 [nM]
Trastuzumab	0.8
**Trz‐LC:p67**	2.6
**Trz‐HC:p67**	4.6
**Trz‐LC:p67‐MMAE**	5.2
**Trz‐HC:p67‐MMAE**	5.4

### 2.3 Cellular Proliferation Assays

**Figure 4:**
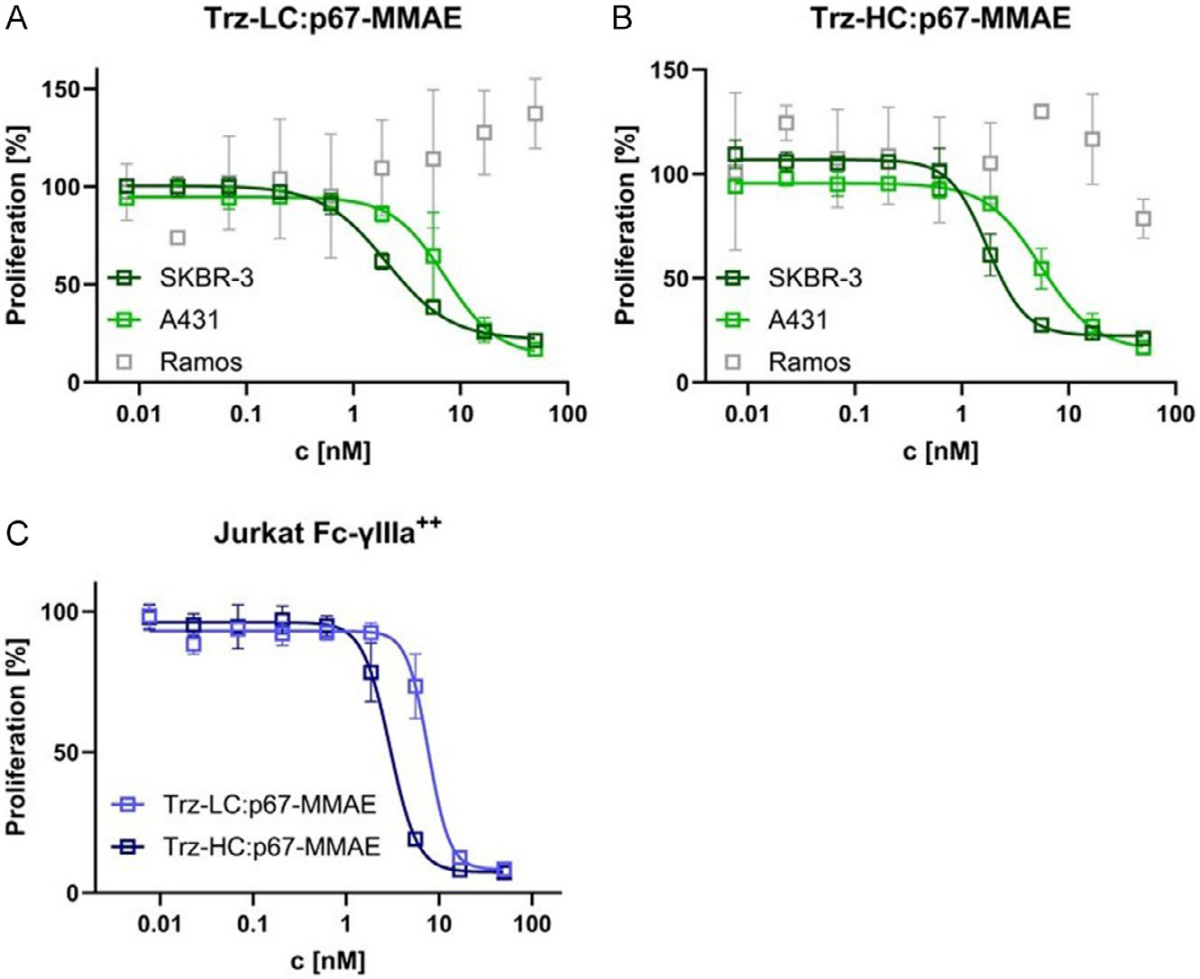
Cellular proliferation assays. A) Assays of **Trz‐LC:p67‐MMAE** conducted on SK‐BR‐3, A431, and Ramos cells. B) Assays of **Trz‐HC:p67‐MMAE** conducted on SK‐BR‐3, A431, and Ramos cells. C) Assays of **Trz‐LC:p67‐MMAE** and **Trz‐HC:p67‐MMAE** conducted on high Fc‐γIIIa expressing Jurkat cells. Technical triplicates are shown.

As cellular binding assays revealed preserved cell binding behavior for **Trz‐LC:p67‐MMAE** and **Trz‐HC:p67‐MMAE**, the ADCs where tested in cellular proliferation assays using cell lines with different Her2‐expression levels (**Figure** **4**A,B). On SK‐BR‐3 cells, both constructs exhibited EC50 values of ≈2 nM (**Table** **2**). These observations apparently do not meet the expectations of subnanomolar EC50 values as reported in literature for MMAE‐loaded trastuzumab ADCs.^[^ ^25^ ^]^ Since cellular binding behavior of the p67 fusion antibodies differed from the one of trastuzumab itself in a similar magnitude, the decreased binding affinity presumably led to a decreased potency compared to the expected subnanomolar EC50. On A431 cells with low Her2‐expression, EC50 values approximately three times higher than the ones on SK‐BR‐3 were observed for both conjugates. On Her2‐negative Ramos cells, no inhibition of cell growth was reported as was to be expected. Trastuzumab is able to recruit immune cells to tumor cells by binding to Fc‐γ receptors via its Fc part. To assess the effect of p67 fusion on Fc‐γ receptor recognition, high Fc‐γIIIa receptor expressing, Her2 negative Jurkat cells were treated with the constructs. Here, a difference in potency was observed, with **Trz‐HC:p67‐MMAE** displaying higher potency than **Trz‐LC:p67‐MMAE** (Figure 4C). As for **Trz‐LC:p67,** the p67 domain was fused to the C‐terminus of the light chain, recognition of Fc‐γIIIa receptor by the antibody Fc might be slightly impaired by sterical hindrance.

**Table 2:** EC50 values and maximum killing derived from cellular proliferation assays of **Trz‐LC:p67‐MMAE** and **Trz‐HC:p67‐MMAE**.

Cell Line	Construct	EC50 [nM]	Max. Killing [%]
SK‐BR‐3	**Trz‐LC:p67‐MMAE**	2.0	78.2 ± 4.2
	**Trz‐HC:p67‐MMAE**	1.7	77.6 ± 4.6
A431	**Trz‐LC:p67‐MMAE**	7.2	86.6 ± 10.5
	**Trz‐HC:p67‐MMAE**	5.5	84.7 ± 8.5
Ramos	**Trz‐LC:p67‐MMAE**	–	–
	**Trz‐HC:p67‐MMAE**	–	–
Jurkat Fc‐γIIIa^++^	**Trz‐LC:p67‐MMAE**	7.6	91.7 ± 8.0
	**Trz‐HC:p67‐MMAE**	3.0	92.6 ± 4.2

## 3 Conclusion

In order to facilitate the generation of ADCs by employing biotin ligase derived from *P. horikoshii*, trastuzumab was equipped with a human‐derived biotin acceptor domain at the C‐terminus of its light chain or heavy chain, respectively. The resultant fusion antibodies, **Trz‐LC:p67** and **Trz‐HC:p67**, were readily produced and purified, exhibiting no aggregation and maintained stability. In a modular chemoenzymatic approach, desthiobiotin azide was conjugated to the p67 domain utilizing PhBL. Subsequent SPAAC with DBCO‐AF488 or DBCO‐MMAE yielded the respective conjugates **Trz‐LC:p67‐AF488**, **Trz‐HC:p67‐AF488**, **Trz‐LC:p67‐MMAE,** and **Trz‐HC:p67‐MMAE**. Successful generation of intermediates and ADCs was monitored by HIC and SDS‐PAGE, also demonstrating high specificity of PhBL ligation. The low amount of unspecific labeling could be reduced by further optimization of reaction conditions, particularly incubation time and buffer conditions. The fusion proteins as well as the final ADCs exhibited single‐digit nanomolar EC50 values in cellular binding assays. A lower EC50 value in on‐cell binding was obtained for unmodified trastuzumab, indicating that the introduction of p67 to the mAb may cause slightly reduced affinity of the fusion antibodies. Whether this also translates into the generation of ADCs other than trastuzumab remains to be elucidated. In cellular proliferation assays, **Trz‐LC:p67‐MMAE** and **Trz‐HC:p67‐MMAE** exhibited low similar single‐digit nanomolar EC50 values on SK‐BR‐3 and threefold EC50 values on A431 cells. As no significant difference in potency was observed between both constructs, the locus of p67 fusion had no impact on the antiproliferative properties of the conjugates. Furthermore, selectivity of the ADCs was proven by absence of antiproliferative effects on Her2‐negative Ramos cells. On a cell line with high Fc‐γIIIa receptor expression, **Trz‐HC:p67‐MMAE** demonstrated higher potency compared to **Trz‐LC:p67‐MMAE**, likely attributable to a moderate blocking of receptor recognition by the C‐terminally fused p67 domain of **Trz‐LC:p67**. However, as the utilized cells were engineered to display artificially high receptor expression and no effect was observed on Fc receptor expressing Ramos cells,^[^ ^26^ ^]^ no off‐tumor issues should arise. Additionally, Fcγ receptor‐dependent internalization of ADCs on‐cell lines with no artificially increased receptor levels cells leading to off‐target cytotoxicity was shown to happen only in the presence of aggregates.^[^ ^27^ ^]^

To summarize, we successfully introduced PhBL as a novel enzyme to expand the toolbox for bioorthogonal generation of ADCs making use of a fully human natural recognition tag for incorporation of modified biotin. Parental fusion antibodies and obtained conjugates maintained desirable hydrophilicity, cell binding, and efficiently mediated cell killing. These studies may pave the way for the generation of ADCs with lowered immunogenic risks and the generation of ADCs with different payloads using a combination of orthogonal ligation enzymes such as biotin ligase, and for example, transglutaminase.

## Conflict of Interest

The authors declare no conflict of interest.

## Supporting information

Supplementary Material

## Data Availability

The data that support the findings of this study are available from the corresponding author upon reasonable request.
