# Supplementary material for: A Recognition Tag of Human Origin for Bioorthogonal Generation of Antibody‐Drug Conjugates using Microbial Biotin Ligase
Source: Chembiochem. 2025 May 6;26(10):e202500261. doi: 10.1002/cbic.202500261 (PMC12117412; doi:10.1002/cbic.202500261)
Supplement: Supplementary file 1 — Supplementary Material [file CBIC-26-e202500261-s001.pdf]

Supplementary Information

A Recognition Tag of Human Origin for Bioorthogonal Generation of Antibody-Drug Conjugates using Microbial Biotin Ligase

Peter Bitsch, [a] Sebastian Bitsch, [a] Noah Murmann, [a] Ingo Bork, [a] Janine Becker[a] and Harald Kolmar*[a],[b]

[a] Peter Bitsch, Dr. Sebastian Bitsch, Noah Murmann, Ingo Bork, Janine Becker, Prof. Dr. Harald Kolmar
Clemens-Schöpf Institute for Organic Chemistry and Biochemistry
Technical University of Darmstadt
Peter-Grünberg-Str. 4, 64287 Darmstadt, Germany
E-mail: Harald.Kolmar@TU-Darmstadt.de

[b] Prof. Dr. Harald Kolmar
Centre of Synthetic Biology
Technical University of Darmstadt
Peter-Grünberg-Str. 4, 64287 Darmstadt, Germany
E-Mail: Harald.Kolmar@TU-Darmstadt.de

Table of Contents

1. Material and Methods **2**
1.1. Materials **2**
1.2. Methods **2**
1.2.1. Hydrophobic interaction chromatography (HIC) 2
1.2.2. Size exclusion chromatography (SEC) 2
1.2.3. Reversed-phase chromatography (RP) 2
1.2.4. Liquid Chromatography-Mass Spectrometry (LC-MS) 2
1.2.5. Photometric measurements for determination of protein concentration and labeling ratio 3
1.2.6. Nuclear magnetic resonance (NMR) spectrometry 3
2. Analytical Data **3**
2.1. Characterization of antibodies **3**
2.1.1. Size-exclusion chromatograms 3
2.2. Western blot of Trz-HC:p67 **4**
2.3. HIC chromatograms of Trz-LC:p67 and Trz-HC:p67 after protein A and Strep-Tactin purification **4**
3. Experimental section **4**
3.1. Sequence of *Pyrococcus horikoshii* biotin ligase (PhBL) **4**
3.2. Sequences of trastuzumab variants **4**
3.2.1. Trastuzumab 4
3.2.2. Trastuzumab-LC:p67 5
3.2.3. Trastuzumab-HC:p67 5
3.3. Production and purification of *Pyrococcus horikoshii* biotin ligase (PhBL) **5**
3.4. Production and purification of trastuzumab variants **6**
3.5. Strep-Tactin purification of trastuzumab variants **6**
3.6. Sodium dodecyl sulfate-polyacrylamide gel electrophoresis (SDS-PAGE) **6**
3.7. Western blot **6**
3.8. Generation of Tras-HC-p67-MMAE and Tras-LC-p67-MMAE conjugates **7**
3.9. Cellular binding assays cells **7**
3.10. Cellular viability assays **7**

3.11. Synthesis of 5-(azidomethyl)-2-oxo-4-imidazolidinehexanoic acid (desthiobiotin azide) 8

1. Material and Methods

1.1. Materials

Solvents were obtained from Acros Organics (Taufkirchen, Germany), Sigma-Aldrich (Darmstadt, Germany) and Thermo Fisher Scientific (Waltham, MA, USA) with the following quality: dichloromethane (DCM), dimethyl sulfoxide (DMSO): synthesis grade; acetonitrile (MeCN), dimethylformamide (DMF): HPLC grade. For HPLC, Millipore quality water was used. All solvents were used without further purification or drying.

Reagents were obtained from Carl Roth GmbH & Co. KG, Thermo Fisher Scientific (Waltham, MA, USA), Sigma-Aldrich (Darmstadt, Germany) and Iris Biotech GmbH (Marktredwitz, Germany). Reagents were used without further purification or drying. DBCO-AF488 was obtained from Lumiprobe (Hannover, Germany), DBCO-Val-Cit-PAB-MMAE was obtained from Merck (Darmstadt, Germany).

1.2. Methods

1.2.1. Hydrophobic interaction chromatography (HIC)

For hydrophobic interaction chromatography (HIC), an Agilent 1260 infinity series device (Agilent, Santa Clara, CA, USA) with variable wavelength detector (VWD) was used with a TSKgel Butyl-NRP (4.6 × 35 mm, 2.5 µM) column (Tosoh Bioscience, Griesheim, Germany). Absorbance was measured at 220 nm. Eluents consisted of eluent A (1.5 M $(NH_4)_2SO_4$, 25 mM tris(hydroxymethyl)aminomethane (Tris), pH = 7.5) and eluent B (25 mM Tris, pH = 7.5). Flow rate was set at 0.7 mL/min. Two and a half minutes of isocratic flow (0% eluent B) was followed by a 25 min gradient flow to reach 100% B. After that, the column was washed with 100% eluent B for two and a half minutes and subsequent isocratic flow 0% B for five minutes.

1.2.2. Size exclusion chromatography (SEC)

For size exclusion chromatography (SEC), an Agilent 1260 infinity series device (Agilent, Santa Clara, CA, USA) with variable wavelength detector (VWD) was used with a TSKgel SuperSW3000 (4.6 × 300 mm, 4 µM) column (Tosoh Bioscience, Griesheim, Germany). Absorbance was measured at 220 nm. Eluent consisted of 1 × PBS at pH = 7.5. Flow rate was set to 0.35 mL/min for 20 min.

1.2.3. Reversed-phase chromatography (RP)

For reversed-phase chromatography (RP), an Agilent LC 1100 series device (Agilent, Santa Clara, CA, USA) with diode array detector (DAD) and an Agilent Eclipse Plus C18 (100 x 4.6 mm, 3.5 µm, 95 Å) column (Agilent, Santa Clara, CA, USA). Absorbance was measured at 220 and 280 nm. Eluents consisted of Eluent A (0.1 % (v/v) TFA in LC-MS-grade water) and eluent B (0.1 % (v/v) TFA in 90 % LC-MS-grade MeCN/water). Three minutes of isocratic flow (starting concentration eluent B) was followed by 20 minutes gradient flow. After that, the column was washed with 100 % eluent B for four minutes and subsequent isocratic flow with starting concentration of eluent B for five minutes.

1.2.4. Liquid Chromatography-Mass Spectrometry (LC-MS)

For LC-MS analysis, a Shimadzu LCMS-2020 mass spectrometer (Shimadzu, Kyōto, Japan) equipped with a Phenomenex Synergy 4 u Fusion-RP 80 (C-18, 250 x 4.6 mm, 2 µm, 80 Å) column (Phenomenex, Torrace, CA, USA) was used. Eluents consisted of Eluent A (0.1 % (v/v) formic acid in LC-MS-grade water) and eluent B (0.1 % (v/v) formic acid in 100 % LC-MS-grade MeCN). The measured unit was the extinction (E) at 220 and 280 nm.

1.2.5. Photometric measurements for determination of protein concentration and labeling ratio

Protein concentration was determined by absorbance measurements carried out with a NanoDrop One (Thermo Fisher Scientific, Waltham, MA, USA) or BioSpec-nano^{TM} (Shimadzu, Kyōto, Japan). For determination of labeling ratios, the devices' programs were used.

1.2.6. Nuclear magnetic resonance (NMR) spectrometry

^{1}H-NMR spectrometry was performed utilizing an Ascend 500 MHz Magnet Avance III Neo Console (Bruker coperation, Billerica, MA, USA) equipped with a 5 mm broad band FO sample head (Bruker coperation, Billerica, MA, USA) with z-gradient SampleCase^{TM} sampler (Bruker coperation, Billerica, MA, USA).

^{13}C-NMR spectrometry was performed utilizing Ascend 300 MHz Magnet Avance III console (Bruker coperation, Billerica, MA, USA) equipped with a 5 mm broad band O sample head (Bruker coperation, Billerica, MA, USA) with z-gradient SampleCase^{TM} sampler (Bruker coperation, Billerica, MA, USA).

Chemical shifts were expressed downfield shifted as delta (δ)-values in parts per million (ppm) corresponding to signals obtained from tetramethylsilane (TMS) and residual signals of proton of the deuterated solvents, respectively.

2. Analytical Data

2.1. Characterization of antibodies

2.1.1. Size-exclusion chromatograms

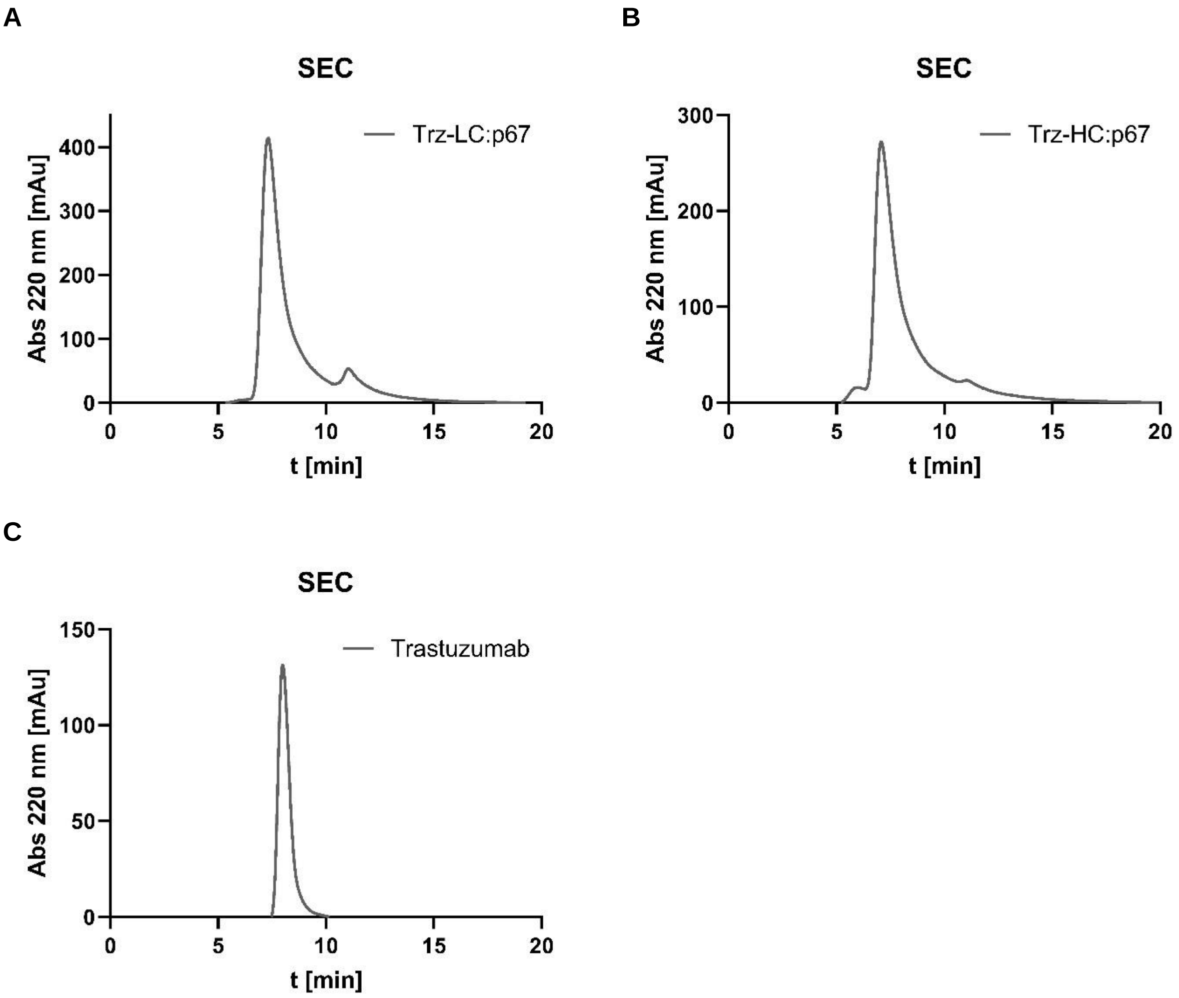

Figure S 1 Size-exclusion chromatograms of produced antibodies. **A**: chromatogram of Trz-LC:p67. **B**: chromatogram of Trz-HC:p67. **C**: chromatogram of trastuzumab.

2.2. Western blot of Trz-HC:p67

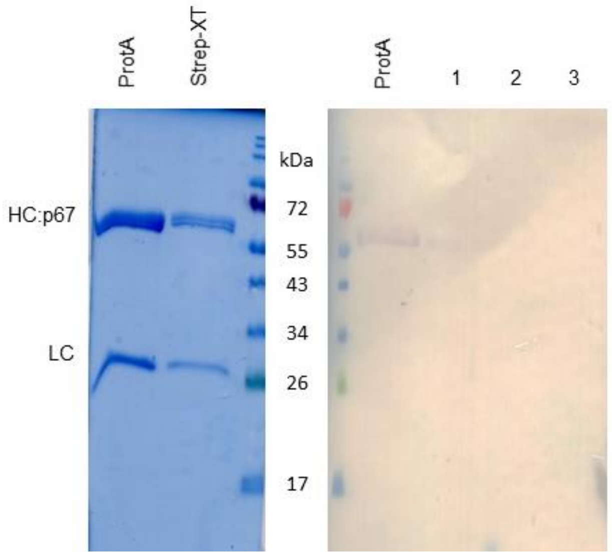

Figure S 2 Coomassie stain (left) and Western blot (right) of Trz-HC:p67 after protein A purification and three consecutive purification steps utilizing Strep-Tactin®XT 4Flow® resin for capturing biotinylated antibody.

2.3. HIC chromatograms of Trz-LC:p67 and Trz-HC:p67 after protein A and Strep-Tactin purification

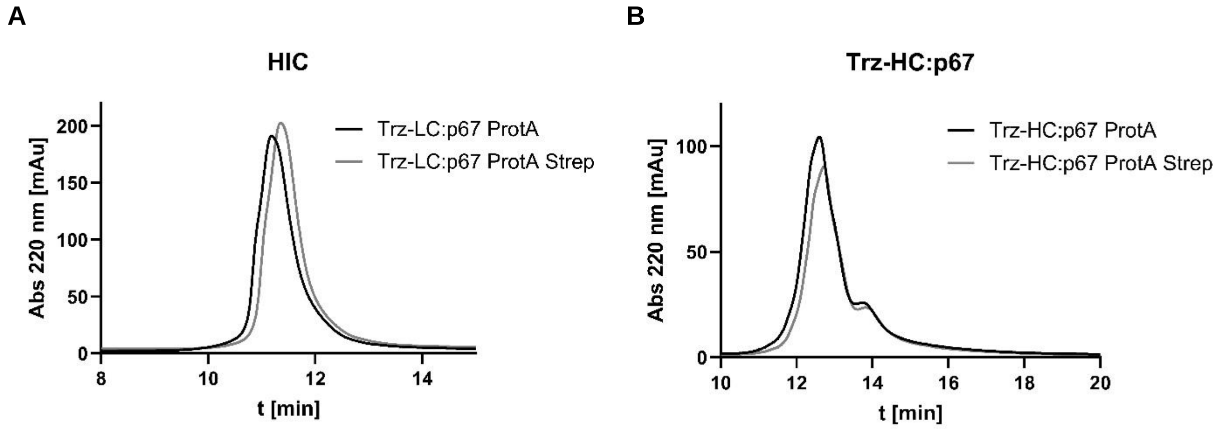

Figure S 3 HIC chromatograms of (**A**) Trz-LC:p67 and (**B**) Trz-HC:p67 after protein A and Strep-Tactin purification (3x, section 3.5).

3. Experimental section

3.1. Sequence of *Pyrococcus horikoshii* biotin ligase (PhBL)

MLGLKTSIIGRRVIYFQEITSTNEFAKTSYLEEGTVIVADKQTMGHGRLNRKWESPEGGLWLSIVLSPKVPQKDLPKIVFLGAV
GVVETLKEFSIDGRIKWPNDVLVNYKKIAGVLVEGKGDKIVLGIGLNVNNKVPNGATSMKLELGSEVPLLSVFRSLITNLDRLY
LNFLKNPMDILNLVRDNMILGVRVKILGDGSFEGIAEDIDDFGRLIIRLDSGEVKKVIYGDVSLRFLHHHHHH

3.2. Sequences of trastuzumab variants

3.2.1. Trastuzumab

HC:

EVQLVESGGGLVQPGGSLRLSCAASGFNIKDTYIHWVRQAPGKGLEWVARIYPTNGYTRYADSVKGRFTISADTSKNTAYL
QMNSLRAEDTAVYYCSRWGGDGFYAMDYWGQGTLVTVSSASTKGPSVFPLAPSSKSTSGGTAALGCLVKDYFPEPVTVS

WNSGALTSGVHTFPAVLQSSGLYSLSSVVTVPSSSLGTQTYICNVNHKPSNTKVDKKVEPPKSCDKTHTCPPCPAPELLGG
PSVFLFPPKPKDTLMISRTPEVTCVVVDVSHEDPEVKFNWYVDGVEVHNAKTKPREEQYNSTYRVVSVLTVLHQDWLNGK
EYKCKVSNKALPAPIEKTISKAKGQPREPQVYTLPPSRDELTKNQVSLTCLVKGFYPSDIAVEWESNGQPENNYKTTPPVLD
SDGSFFLYSKLTVDKSRWQQGNVFSCSVMHEALHNHYTQKSLSLSPG
LC:
DIQMTQSPSSLSASVGDRVTITCRASQDVNTAVAWYQQKPGKAPKLLIYSASFLYSGVPSRFSGSRSGTDFTLTISSLQPED
FATYYCQQHYTTPPTFGQGTKVEIKRTVAAPSVFIFPPSDEQLKSGTASVVCLLNNFYPREAKVQWKVDNALQSGNSQESV
TEQDSKDSTYSLSSTLTLSKADYEKHKVYACEVTHQGLSSPVTKSFNRGEC

3.2.2. Trastuzumab-LC:p67

HC:
EVQLVESGGGLVQPGGSLRLSCAASGFNIKDTYIHWVRQAPGKGLEWVARIYPTNGYTRYADSVKGRFTISADTSKNTAYL
QMNSLRAEDTAVYYCSRWGGDGFYAMDYWGQGTLVTVSSASTKGPSVFPLAPSSKSTSGGTAALGCLVKDYFPEPVTVS
WNSGALTSGVHTFPAVLQSSGLYSLSSVVTVPSSSLGTQTYICNVNHKPSNTKVDKKVEPPKSCDKTHTCPPCPAPELLGG
PSVFLFPPKPKDTLMISRTPEVTCVVVDVSHEDPEVKFNWYVDGVEVHNAKTKPREEQYNSTYRVVSVLTVLHQDWLNGK
EYKCKVSNKALPAPIEKTISKAKGQPREPQVYTLPPSRDELTKNQVSLTCLVKGFYPSDIAVEWESNGQPENNYKTTPPVLD
SDGSFFLYSKLTVDKSRWQQGNVFSCSVMHEALHNHYTQKSLSLSPG
LC:
DIQMTQSPSSLSASVGDRVTITCRASQDVNTAVAWYQQKPGKAPKLLIYSASFLYSGVPSRFSGSRSGTDFTLTISSLQPED
FATYYCQQHYTTPPTFGQGTKVEIKRTVAAPSVFIFPPSDEQLKSGTASVVCLLNNFYPREAKVQWKVDNALQSGNSQESV
TEQDSKDSTYSLSSTLTLSKADYEKHKVYACEVTHQGLSSPVTKSFNRGECGGGGSGGGGSGGGGSLRSPMPGVVVAVS
VKPGDAVAEGQEICVIEAMKMQNSMTAGKTGTVKSVHCQAGDTVGEGDLLVELE

3.2.3. Trastuzumab-HC:p67

HC:
EVQLVESGGGLVQPGGSLRLSCAASGFNIKDTYIHWVRQAPGKGLEWVARIYPTNGYTRYADSVKGRFTISADTSKNTAYL
QMNSLRAEDTAVYYCSRWGGDGFYAMDYWGQGTLVTVSSASTKGPSVFPLAPSSKSTSGGTAALGCLVKDYFPEPVTVS
WNSGALTSGVHTFPAVLQSSGLYSLSSVVTVPSSSLGTQTYICNVNHKPSNTKVDKKVEPPKSCDKTHTCPPCPAPELLGG
PSVFLFPPKPKDTLMISRTPEVTCVVVDVSHEDPEVKFNWYVDGVEVHNAKTKPREEQYNSTYRVVSVLTVLHQDWLNGK
EYKCKVSNKALPAPIEKTISKAKGQPREPQVYTLPPSRDELTKNQVSLTCLVKGFYPSDIAVEWESNGQPENNYKTTPPVLD
SDGSFFLYSKLTVDKSRWQQGNVFSCSVMHEALHNHYTQKSLSLSPGLRSPMPGVVVAVSVKPGDAVAEGQEICVIEAMK
MQNSMTAGKTGTVKSVHCQAGDTVGEGDLLVELE
LC:
DIQMTQSPSSLSASVGDRVTITCRASQDVNTAVAWYQQKPGKAPKLLIYSASFLYSGVPSRFSGSRSGTDFTLTISSLQPED
FATYYCQQHYTTPPTFGQGTKVEIKRTVAAPSVFIFPPSDE**C**LKSGTASVVCLLNNFYPREAKVQWKVDNALQSGNSQESV
TEQDSKDSTYSLSSTLTLSKADYEKHKVYACEVTHQGLSSPVTKSFNRGEC

3.3. Production and purification of *Pyrococcus horikoshii* biotin ligase (PhBL)

The gene was cloned into a pET30 expression vector and expressed in *E. coli* cells. Expression of proteins was carried out in *E. coli* BL21 (DE3). One day before expression, an overnight culture was prepared by inoculating cells in 50 ml dYT with 75 µg/mL kanamycin. This culture was used to inoculate 1 L of TB-medium with 75 µg/mL kanamycin to an OD_{600} of 0.1. The main culture was incubated at 37°C and 200 rpm until OD_{600} reached ~ 0.6. Protein expression was induced by the addition of 0.5 mM (final) IPTG and was carried out overnight at 25°C. The next day, cells were harvested by centrifugation at 4000 × g. The centrifugate was either stored at -80°C or was directly used for purification.

Cell centrifugate was resuspended in 25 ml of IMAC A buffer (20 mM imidazole, 20 mM Tris, 300 mM NaCl, pH 7.5). Sonification procedure was carried out at ~50 % of maximal power five times with one minute pause in between. Subsequently, the lysate was centrifugated at 15000 × g for 20 min at 4°C and the supernatant was filtered with an 0.45 µm syringe filter.

His-Tag IMAC was performed utilizing an ÄKTA Start (GE Healthcare Life Science, Uppsala, Sweden)) equipped with a HisTrap HP purification column (GE Healthcare Life Science). IMAC A served as binding and washing buffer; IMAC B (500 mM imidazole, 20 mM Tris, 300 mM NaCl, pH 7.5) was used for elution. After elution, proteins were transferred into a dialysis tube with a molecular weight cut off of 6000–8000 and incubated overnight at 4 °C in 5 L 50 mM Tris pH 7.5, 150 mM NaCl and 5 mM $MgCl_2$.

3.4. Production and purification of trastuzumab variants

Expi HEK293F cells were transfected with pTT5 plasmids of respective HC and LC variants. Cells were inoculated in Expi293 expression medium 37 °C and 8 % CO_2 at 110 rpm. After 5 days, culture broth was centrifugated at 12000× g and supernatant was filtrated. Filtrate was purified utilizing an ÄKTAstart (GE Healthcare Life Science, Uppsala, Sweden) equipped with a ProteinA HP column (GE Healthcare Life Science), following the instructions of the supplier. 1× PBS served as binding and washing buffer; 0.1 M citric acid at pH = 3 was used for elution. Eluate was collected in microtubes with 100 µL 1 M Tris-HCl at pH = 9.0 added per mL of eluate. After elution, proteins were transferred into a dialysis tube with a molecular weight cut off of 30000 g/mol and incubated overnight at 4 °C in 5 L of 1× PBS.

3.5. Strep-Tactin purification of trastuzumab variants

A 2 mL syringe was filled with 400 µL of Strep-Tactin®XT 4Flow® resin. Resin was prepared for sample application following the instructions of the supplier. The protein solution was applied to the resin and flow through immediately collected as it contained the unbiotinylated antibody. The flow through was applied to the resin two additional times, proceeding as before. Samples of each application step were collected and analyzed by SDS-PAGE and Western blot.

3.6. Sodium dodecyl sulfate-polyacrylamide gel electrophoresis (SDS-PAGE)

Acrylamide SDS-gels were prepared with 15 % acrylamide in separation and 4 % in stacking gel. Composition of the SDS-gel can be found in **Table S 1** For sample preparation, SDS-loading dye was added to the protein solution ($m_{protein}$ = 5µg) and the sample was heated to 98°C for 5 min. Samples were loaded to the gel and proteins were separated applying 300 V and 45 mA until the dye front reached the end of the gel. Subsequently, the gel was stained with Coomassie brilliant blue or a western blot was performed.

Table S 1: Composition of utilized SDS-gels.

Compound	Stacking gel 4 %	Separation gel 15 %
Rotiphorese 40 (37.5:1)	2.4 ml	13 ml
Separation Tris buffer	-	6.5 ml
Stacking Tris buffer	3.6 ml	-
dH_2O	8.4 ml	6.5 ml
TEMED	10.8 µl	9.8 µl
APS 10 % (w/v)	130 µl	195 µl

3.7. Western blot

An unstained acrylamide SDS-gel was blotted onto a nitrocellulose membrane by application of a voltage of 25 V and amperage of 300 mA for 35 min. Subsequently, the membrane was blocked to saturation with 3 % of skim milk powder in PBS-T for 1 h. Subsequently, streptavidin alkaline phosphatase conjugate (Roche, 11089161001) was applied for 1h in 15 ml PBS-T (1:5000), followed by three times washing of the membrane with PBS-T and equilibration of the membrane in AP-buffer (100 mM Tris-HCl pH 9.1, 100 mM NaCl, 5 mM $MgCl_2$). Staining was performed with 15 ml AP-buffer containing 75 µL 5-Bromo-4-chloro-3-indolyl phosphate (BCIP) (50 mg/mL in DMF) and 25 µL nitro blue tetrazolium chloride (NBT) (75 mg/mL in 70 % DMF) until protein bands were visible. Reaction was quenched utilizing 10 % acetic acid.

3.8. Generation of Tras-HC-p67-MMAE and Tras-LC-p67-MMAE conjugates

20 molar equivalents of 5-(azidomethyl)-2-oxo-4-imidazolidinehexanoic acid and 0.1 molar equivalents of biotin ligase were added to a solution of 0.5 mg trastuzumab-p67 variants (c > 5mg/mL) in 5 mM Magnesium acetate ($Mg(OAc)_2$), 2.5 mM ATP and 50 mM bicine buffer and incubated at 37 °C for 3 hours. Subsequently, conjugates were purified utilizing Protein A HP SpinTrap™ columns (Cytiva Europe GmbH, Freiburg, Germany) following the instructions of the supplier.

Subsequently, 6 molar equivalents of drug-linker were added as well as DMSO to a final 25% v/v of DMSO and incubated at 30°C for 18 hours. Conjugates were purified utilizing Pur-A-Lyzer™ Mini Dialysis Kit (Merck KGaA Darmstadt, Germany) following the instructions of the supplier in two dialysis steps, performing the buffer exchange after 4 hours. Reaction progress was monitored with HIC analysis, product characterization was performed with HIC, SEC and SDS-PAGE (15 % acrylamide).

3.9. Cellular binding assays cells

Cell lines utilized for binding was SK-BR-3, known to overexpress Her2,[25] 50000 cells per well were incubated with dilution rows of trastuzumab, Trz-LC:p67, Trz-HC:p67, Trz-LC:p67-MMAE and Trz-HC:p67-MMAE, respectively, in binding buffer (1× PBS with 0.1% m/v bovine serum albumin) in a U-bottom 96-well plate for 30 min at 4 °C. After washing with binding buffer, 7 µL of a 1:80 dilution of Goat anti-Human IgG Fc secondary antibody, PE, eBioscience™ (AB_465926, ThermoFisher Scientific, Darmstadt, Germany) was added to each well for staining and cells were incubated for additional 20 min at 4 °C. Subsequently, cells were washed with binding buffer and analyzed in the cytometer (CytoFLEX, Beckman Coulter GmbH, Krefeld, Germany). For data analysis, mean FI was calculated and plotted against concentration. Assay was conducted in technical triplicates. Experiments were conducted in at least three biological replicates with three technical replicates each.

3.10. Cellular viability assays

6000 cells/well were seeded in a U bottom 96 well plate in 90 µL of medium (**Table S 2**) and incubated over night at 37 °C and 5% CO_2. Jurkat cells utilized for this assay were derived from mechanism-of-action (MOA)-based bioassay core kit to measure FcγRIIIa (V158) function (Promega GmbH, Walldorf, Germany). The next day, 10 µL of a dilution row of each sample was added and cells were incubated for 3 days at 37°C and 5% CO_2. To evaluate viability, cellular viability assay was performed utilizing CellTiter-Blue® (Promega GmbH, Walldorf, Germany) following the instructions if the supplier. Fluorescence measurement was conducted with a CLARIOstar Plus (BMG Labtech, Ortenberg, Germany) plate reader. Experiments with SKBR3, A431 and Ramos were conducted in at least two biological replicates with three technical replicates each. Assays on Jurkat cells were performed in technical triplicates.

Table S 2 Cell lines and corresponding media utilized for cellular viability assays.

Cell line	Medium
SK-BR-3	90% DMEM, 10% FBS
A431	90% DMEM, 10% FBS
Ramos	90% RPMI 1640 + 10% FBS
Jurkat (Fc-γIIIa^{++})	90% RPMI 1640 + 10% FBS

3.11. Synthesis of 5-(azidomethyl)-2-oxo-4-imidazolidinehexanoic acid (desthiobiotin azide)

5-(azidomethyl)-2-oxo-4-imidazolidinehexanoic acid (desthiobiotin azide, **1**) was synthesized as reported previously.[20] The product was obtained as colorless oil (4.615 mg).

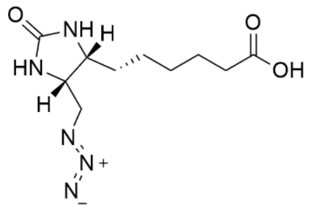

Molecular Weight: 255,27

**LC-MS** (ESI+):	*m/z* (found)	256.38, 511.37 t_R = 10.35-10.85 min.
	m/z (calc.)	256.27 $(M+H)^+$, 511.54 $(M+2H)^{2+}$

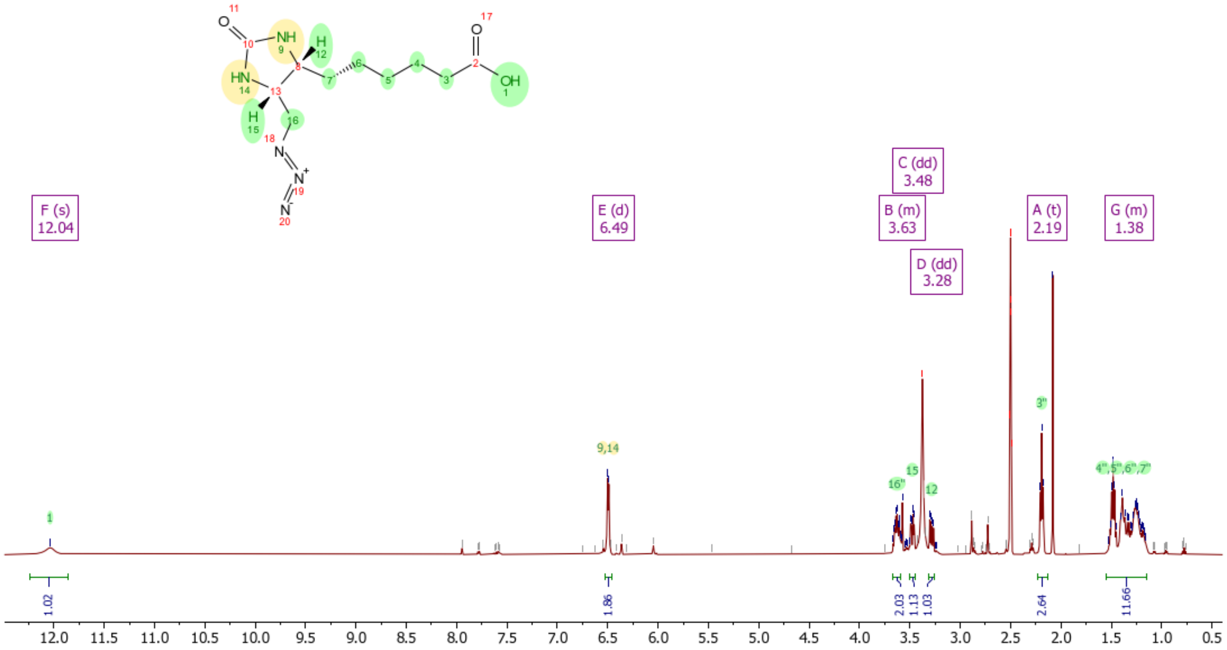

NMR spectrum 1: ^{1}H NMR spectrum (500 MHz, DMSO-d_6) of **1**.

^{1}H NMR (DMSO-*d6*, 500 MHz): δ 12.04 (bs, 1H), 6.49 (s, 2H), 3.63 (m, 2H), 3.48 (dd, J = 12.5, 4.5, 1H), 3.28 (dd, J = 12.5, 6.1, 1H), 2.19 (t, J = 7.4, 2H), 1.14-1.53 (m, 8H).

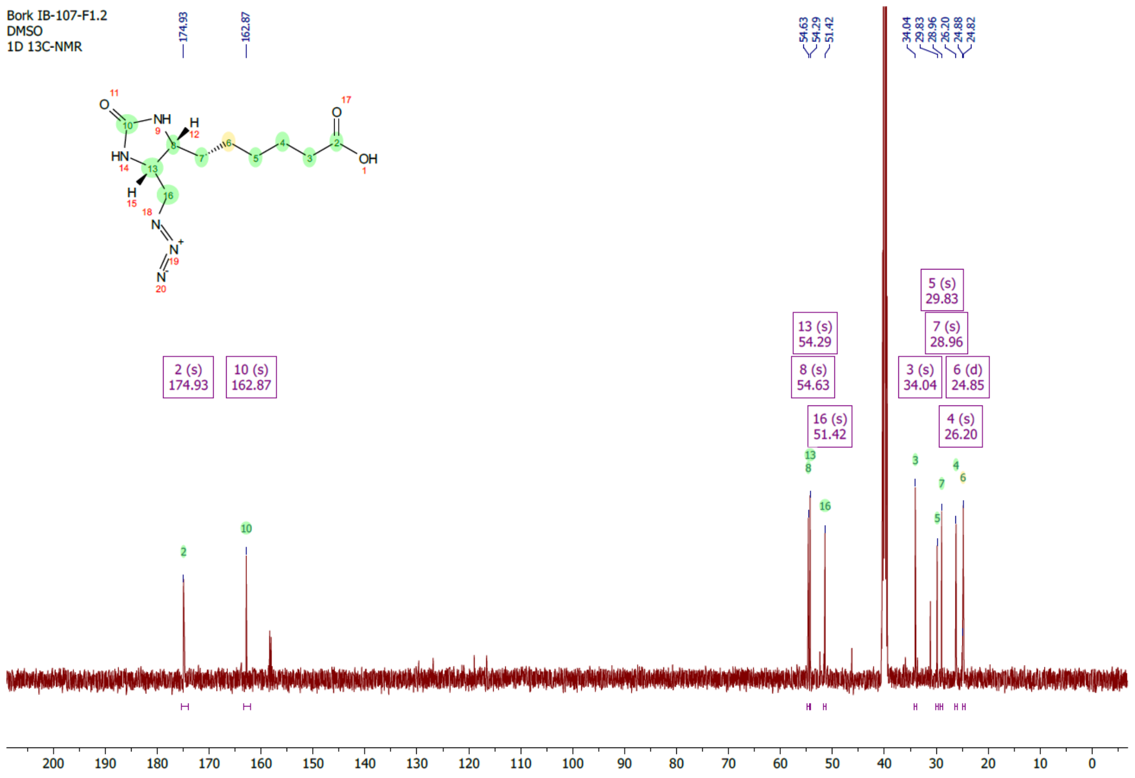

NMR spectrum 2: ^{13}C NMR spectrum (300 MHz, DMSO-d_6) of **1**.

^{13}C NMR (DMSO-*d6*, 75 MHz): δ 174.93, 162.87, 54.63, 54.29, 51.42, 34.04, 29.83, 28.96, 26.20, 24.85.

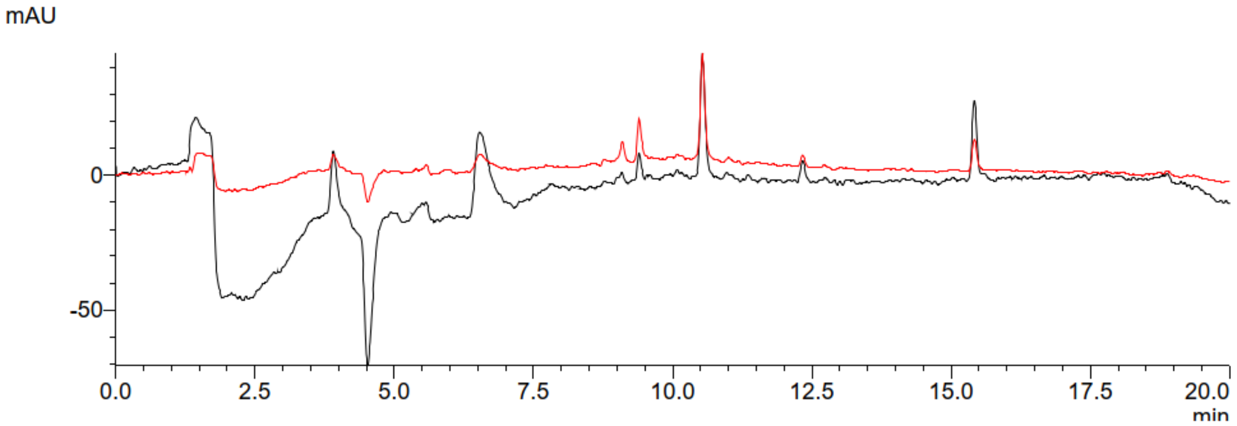

LC-MS chromatogram 1: LC-MS analysis of 5-(azidomethyl)-2-oxo-4-imidazolidinehexanoic acid with detection at 220 nm (black) and 280 nm (red).

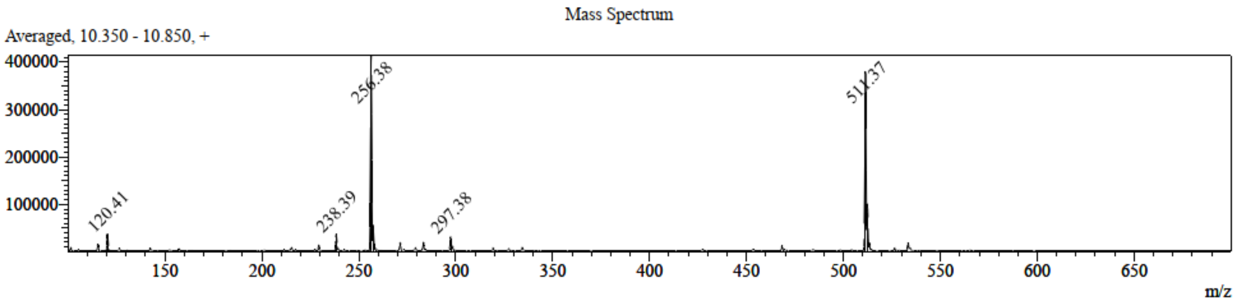

MS spectrum 1: MS-spectrum of 5-(azidomethyl)-2-oxo-4-imidazolidinehexanoic acid (ESI, +).
